# Trend in mortality from mental and behavioral disorders due to alcohol use in Brazil, 2010-2021

**DOI:** 10.1590/S2237-96222024V33E20231483.en

**Published:** 2024-07-15

**Authors:** Wygor Bruno e Silva Morais, Fernando Ferraz do Nascimento, Jardeliny Corrêa da Penha, Jesusmar Ximenes Andrade, Márcio Dênis Medeiros Mascarenhas, Malvina Thais Pacheco Rodrigues

**Affiliations:** 1 Universidade Federal do Piauí, Programa de Pós-Graduação em Saúde e Comunidade, Teresina, PI, Brazil

**Keywords:** Alcohol-Related Disorders, Mortality, Alcoholism, Time Series Studies, Adult Health, Mental Health, Trastornos por Consumo de Alcohol, Mortalidad, Alcoholismo, Estudios de Series Temporales, Salud del Adulto, Salud mental

## Abstract

**Objective:**

To analyze the trend in mortality from mental and behavioral disorders due to alcohol use in Brazil, 2010-2021.

**Methods:**

This was an time series study using Mortality Information System data. Annual percentage change (APC) and 95% confidence intervals (95% CI) were calculated using Prais-Winsten linear regression.

**Results:**

Mortality showed a stationary trend for Brazil as a whole (APC = 0.6; 95%CI -4.2;3.0), a falling trend in individuals aged 20-29 years in the South (APC = -7.4; 95%CI -10.0;-4.3) and Northeast (APC = -3.4; 95%CI -6.4;-0.4) regions, in people aged 30-39 in the Midwest region (APC = -3,8; 95%CI -7.4;-0.1) and 40-49 in the South (APC = -2.1; 95%CI -3.8;-0.4), North (APC = -3.1; 95%CI -5.7;-0.5) and Midwest (APC = -2.9; 95%CI -5.5;-0.3) regions.

**Conclusion:**

Mortality from mental and behavioral disorders due to alcohol use showed a stationary trend nationally and a falling trend in some age groups regionally.

## Study contributions

**Main results:**

The mortality coefficient was highest in the 50-59 year age group. Mortality showed a stationary trend in Brazil as a whole, falling in individuals aged 20-29 (South; Northeast), 30-39 (Midwest) and 40-49 (North; Midwest; South).

**Implications for services:**

Mental and behavioral disorders due to alcohol use affect Brazilians at different stages of life, and it is essential to raise the population’s awareness as to reducing consumption of alcoholic beverages.

**Perspectives:**

The need exists to target actions to promote health and prevent mortality related to alcohol use, with emphasis on the 50-59 age group.

## Resumen

**Objetivo:**

Analizar la tendencia de mortalidad por trastornos mentales y del comportamiento en decorrencia del consumo de alcohol en Brasil en el período 2010-2021.

**Métodos:**

Estudio de series de tiempo ecológicas, con datos del Sistema de Información de Mortalidad; el cambio porcentual anual (APV) y los respectivos intervalos de confianza del 95% (IC_95%_) se calcularon mediante regresión lineal de Prais-Winsten.

**Resultados:**

Hubo una tendencia estacionaria en la mortalidad en Brasil en su conjunto (VPA = 0,6; IC_95%_ -4,2;3,0), una tendencia decreciente en individuos de 20 a 29 años en las regiones del Sur (VPA = -7,4; IC_95%_ -10,0;-4,3) y Noreste (VPA = -3,4; IC_95%_ -6,4;-0,4), en personas de 30 a 39 años en el Centro-Oeste (VPA = -3,8; IC_95%_ -7,4;-0,1) y en adultos de 40 a 49 años en el Sur (VPA = -2,1; IC_95%_ -3,8;-0,4), Norte (VPA = -3,1; IC_95%_ -5,7;-0,5) y Centro-Oeste (VPA = - 2,9; IC_95%_ -5,5;-0,3).

**Conclusión:**

La mortalidad por trastornos mentales y del comportamiento a causa del consumo de alcohol presentó una tendencia estacionaria en Brasil y una tendencia decreciente en algunos grupos etarios.

## INTRODUCTION

Alcohol is a legalized and widely disseminated psychoactive drug, which acts directly on the central nervous system causing chemical and physical dependence.^1^ ^)^ Harmful use of this substance is a public health problem, causing illness, disability and death, and is associated with the appearance of more than 200 diseases and health problems, including mental and behavioral disorders and infectious diseases, such as tuberculosis and HIV. Furthermore, it causes unintentional and intentional injuries, such as those caused by traffic accidents, violence and suicide, resulting in social and economic problems for individuals and society in general.^2^

Every year worldwide, approximately 3 million deaths are due to harmful use of alcohol, accounting for 5.3% of total deaths. Based on calculations of disability-adjusted life years lost, 5.1% of the global burden of disease and injury is attributed to alcohol consumption. With regard to Brazil, where around 6.2% of deaths are related to alcoholic beverage consumption, alcohol ranks third nationally and fourth globally among risk factors for disease burden.^3^

Mental and behavioral disorders associated with excessive alcohol consumption contribute to continuing morbidity and mortality and disabilities resulting from its harmful use,^3^ ^),(^ ^4^ in particular acute intoxication, use that is harmful to health, dependence syndrome, withdrawal syndrome, withdrawal syndrome with delirium or acute confusional state, alcohol-induced psychosis, amnestic disorder, residual or late-onset psychotic disorder, other mental or behavioral disorders and unspecified mental or behavioral disorder.^5^

Brazilian studies on hospitalizations and deaths related to alcohol consumption provide relevant information for understanding its economic and social impacts on the population.^6^ ^)-(^ ^8^ However, mortality due to mental and behavioral disorders is an underexplored topic, lacking studies that assess it among Brazilian adults, these being the main excessive consumers of alcohol in Brazil.

The objective of this study was to analyze the trend in mortality from mental and behavioral disorders due to use of alcohol in Brazil, from 2010 to 2021.

## METHODS

*Design*

This was an time series study, aimed at analyzing the trend in mortality due to mental and behavioral disorders resulting from alcohol use in Brazil, from 2010 to 2021. The units of analysis were the five Brazilian geographic macro-regions: North, Northeast, Southeast, South and Midwest.^4^

*Background*

Brazil, the largest country in Latin America and fifth largest in the world in terms of territorial area, had a population of 203 million inhabitants in August 2022, distributed over 5,571 municipalities in 26 states and the Federal District.

*Participants*

This was study of deaths of adults aged 20 to 59 whose underlying cause of death was “mental and behavioral disorders due to use of alcohol”, occurring in Brazil between 2010 and 2021.

*Variables*

The variables used in this study were:

a) Sociodemographic variables

- sex (male; female);

- age group (in years: 20-29; 30-39; 40-49; 50-59);

- race/skin color (White; Black; mixed race; Asian/Indigenous);

- Brazilian geographic macro-region (North; Northeast; Southeast; South; Midwest);

- year of death, in the period 2010-2021; and

b) Mortality indicator

- coefficient of mortality from mental and behavioral disorders due to use of alcohol.

*Data sources and measurement*

The mortality data were extracted from the Mortality Information System (*Sistema de Informações sobre Mortalidade* - SIM) database, available at the Brazilian Ministry of Health National Health System Information Technology Department website (https://datasus.saude.gov.br/), between June and July 2023.

Data were obtained on deaths that occurred between 2010 and 2021 of people resident in Brazil’s five macro-regions, whose underlying cause of death was classified under code F-10 (Mental and behavioral disorders due to use of alcohol), of the International Statistical Classification of Diseases and Related Health Problems - Tenth Revision (ICD-10). The population data, made available by the Brazilian Institute of Geography and Statistics (*Instituto Brasileiro de Geografia e Estatística* - IBGE), were captured by macro-region, age group and sex, for the period 2000-2021.

In order to calculate coefficients of mortality from mental and behavioral disorders due to use of alcohol, we divided the number of deaths according to place of residence (numerator), in the period studied, by the population in the same period and place of residence (denominator), and then multiplied the quotient by 100,000 inhabitants. These coefficients were calculated by age group, sex, race/skin color and geographic macro-region of Brazil, for each year of death. The arithmetic mean was then calculated, adding together the coefficients for each year and dividing them by the total of 12 years studied. For the purposes of controlling aggregation bias, data on the population studied were stratified by macro-region, sex, age group and race/skin color, so as to achieve better characterization of mortality through a more homogeneous comparison of the population.

*Statistical methods*

The data were grouped into spreadsheets on the Microsoft Excel platform and exported to the Statistical Package for the Social Sciences (SPSS), version 2.0, which we used to perform statistical analyses. We used the Prais-Winsten linear regression model to verify temporal trend, as it is the recommended method for correcting serial autocorrelation and enabling analysis of time series of study periods greater than nine years.^9^ ^)^ The mortality rate for mental and behavioral disorders due to use of alcohol was taken as the dependent variable (Y), according to age group, sex, race/skin color and geographic macro-region; while the independent variable (X) was the year of death, between 2010 and 2021.

We calculated the base 10 logarithm of the Y values and then applied the Prais-Winsten autoregressive model to estimate the β1 coefficient values of the regression equation.

Annual percentage change (APC) was calculated using the following formula:

APC = (-1+10 [b1])*100%

and the respective 95% confidence intervals % (95%CI) were calculated by applying the following formulae:

lower 95%CI = (-1+10[b1-t*e])*100%

upper 95%CI = (-1+10[b1+t*e ])*100%

Adopting a 5% significance level.

Mortality coefficient trends were interpreted as follows: rising trend when APC was significantly positive; falling trend when APC was significantly negative; and stationary trend when there was no significant difference between the APC value and zero.^6^

*Ethical aspects*

This study used secondary data available from official Brazilian Ministry of Health, databases whereby study participants were not identified. Therefore, submission to a Research Ethics Committee was not necessary, in accordance with National Health Council Resolution No. 466, dated December 12, 2012.

## RESULTS

Between 2010 and 2021, Brazil recorded 60,513 deaths from mental and behavioral disorders due to use of alcohol. The Southeast region of the country recorded the highest number of deaths due to this condition (22,910), while the Northern region (2,056) recorded the lowest number of deaths. The majority of deaths corresponded to individuals of the male sex (90.0%), with the highest proportion of individuals in the 50-59 age group (40.6%) and of self-reported mixed race (50.3%) (Table 1).

**Table 1:** Sociodemographic characterization of deaths from mental and behavioral disorders due to use of alcohol in individuals 20-59 years old, Brazil, 2010-2021

Variable	North		Northeast		Midwest		South		Southeast		Brazil	
	N (2,056)	%	N (21,120)	%	N (5,244)	%	N (9,183)	%	N (22,910)	%	N (60,513)	%
Sex												
Male	1,858	90.4	19,260	91.2	4,705	89.7	8,437	92	20,263	88.5	54,523	90.0
Female	198	9.6	1,860	8.8	539	10.3	746	8.0	2,647	11.5	5,990	10.0
Race/skin color												
White	217	10.5	2,691	12.7	1,180	22.5	6,481	70.5	8,408	37.0	18,977	31.4
Mixed race	1,540	75.0	14,660	69.5	3,241	62.0	1,675	18.0	9,333	40.7	30,449	50.3
Black	194	9.5	2,477	11.7	616	11.5	772	8.5	4,044	17.5	8,103	13.3
Asian and Indigenous	41	2.0	108	0.5	107	2.0	69	1.0	70	0.3	395	0.7
Unknown	64	3. 0	1,184	5.6	100	2.0	186	2.0	1,055	4.5	2,589	4.3
Age group (years)												
20-29	122	6.0	1,129	5.3	206	4.0	215	2.0	656	3.0	2,328	3.8
30-39	438	21.5	4,585	21.7	1,078	20.5	1,318	14.5	3,852	17.0	11,271	18.6
40-49	731	35.5	7,767	36.8	1,999	38.0	3,301	36.0	8,519	37.0	22,317	37.0
50-59	765	37.0	7,640	36.2	1,961	37.5	4,349	47.5	9,888	43.0	24,603	40.6

**Figure 1:**
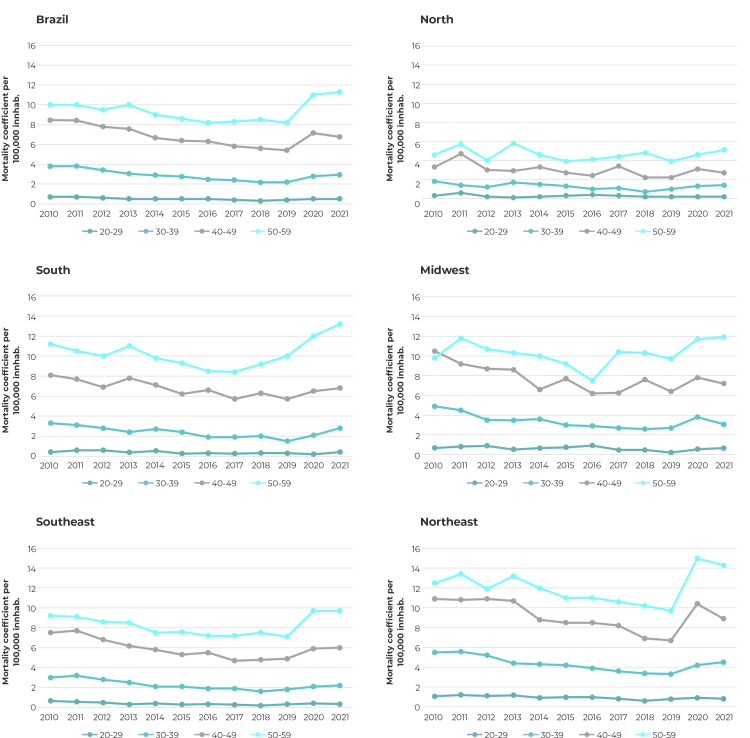
Coefficients of mortality from mental and behavioral disorders due to use of alcohol in individuals 20-59 years old, by national macro-regions, Brazil, 2010-2021

The mortality coeffcient was higher in the 50-59 age group, both for Brazil as a whole and also for each of the country’s macro-regions. In Brazil as a whole, the mortality coefficient for the 50-59 age group ranged from 10.1 to 11.3 per 100,000 inhab. between 2010 and 2021, indicative of a stationary trend (APC = 0.5; 95%CI - 3.1;4.2). The other age groups showed a reduction in the coefficient, both for the entire country and also for each of its regions (Figure 1).

A stationary trend was found in the mortality coefficient for mental and behavioral disorders due to use of alcohol in Brazil as a whole (APC = 0.6; 95%CI -4.2;3.0), in both sexes and in the country’s five regions (Table 2). However, the mortality coefficient trend according to age group differed between regions. It was falling for people aged 20-29 living in the Northeast region (APC = -3.4; 95%CI -6.4;-0.4) and the Southern region (APC = -7.4; 95%CI -10.0;-4.3), while the North (APC = -4.5; 95%CI -11.0;2.4), Southeast (APC = -5.8; 95%CI -11.0;0.7) and Midwest regions (APC = -5.0; 95%CI -11.0;1.4) showed a stationary trend for the same age range. With regard to those aged 30-39, only the Midwest region showed a falling mortality trend for mental and behavioral disorders due to use of alcohol (APC = -3.8; 95%CI -7.4;-0.1). Among individuals aged 40-49, this trend was falling in the North (APC = -3.1; 95%CI -5.7;-0.5), South (APC = -2.1; 95%CI -3.8;-0.4) and Midwest regions (APC = -2.9; 95%CI -5.5;-0.3) (Table 3). Mortality among individuals aged 50-59 showed a stationary trend in all five regions of the country (Figure 1).

**Table 2:** Trends in coefficients of mortality from mental and behavioral disorders due to use of alcohol in individuals 20-59 years old, by geographic macro-region and sex, Brazil, 2010-2021

Variables	Mean coeffcient	APC^a^ (%)	95%CI^b^	p-value^c^	Interpretation^d^
Region					
Brazil	41.4	0.6	(-4.2;3.0)	0.694	Stationary
North	18.2	-1.7	(-3.7;0.4)	0.111	Stationary
Northeast	56.9	-0.8	(-4.2;2.7)	0.606	Stationary
Southeast	38.3	0.8	(-4.7;3.3)	0.670	Stationary
South	45.5	0.1	(-3.7;4.2)	0.928	Stationary
Midwest	48.3	-0.9	(-3.8;1.9)	0.474	Stationary
Male sex					
Brazil	76.5	-0.6	(3.2;-4.2)	0.728	Stationary
North	32.8	2.0	(-4.0;0.0)	0.060	Stationary
Northeast	107.6	0.7	(-4.3;3.0)	0.682	Stationary
Southeast	69.4	-0.6	(-4.7;3.3)	0.741	Stationary
South	85.0	-0.0	(-3.6;3.8)	0.995	Stationary
Midwest	87.8	-0.9	(-4.0;2.2)	0.517	Stationary
Female sex					
Brazil	7.7	-1.7	(-4.2;0.9)	0.173	Stationary
North	3.4	1.2	(-2.0;4.6)	0.435	Stationary
Northeast	9.6	-1.8	(-3.9;0.3)	0.095	Stationary
Southeast	8.6	-2.4	(-4.9;0.1)	0.061	Stationary
South	7.3	-1.8	(-5.7;2.3)	0.335	Stationary
Midwest	9.8	-1.8	(-4.8;1.2)	0.205	Stationary

a) APC: Annual percentage change; b) 95%CI: 95% confidence interval; c) p-value: Significance of coeffcient association using Prais-Winsten *t* test regression analysis; d) Stationary trend when p-value ≥ 0.05.

**Table 3:** Trends in coefficients of mortality from mental and behavioral disorders due to use of alcohol in individuals 20-59 years old, by region and age group, Brazil, 2010-2021

Age group (years)	Mean coefficient	APC^a^ (%)	95%CI^b^	p-value^c^	Interpretation^d^
Brazil					
20-29	5.5	-4.2	(-7.8;-0.2)	0.038	Falling
30-39	27.7	-2.7	(-6.8;1.7)	0.199	Stationary
40-49	65.6	-2.5	(-5.5;0.6)	0.103	Stationary
50-59	91.5	0.5	(-3.1;4.2)	0.762	Stationary
North					
20-29	3.1	-4.5	(-11.0;2.4)	0.174	Stationary
30-39	13.2	-3.1	(-8.1;2.2)	0.222	Stationary
40-49	30.1	-3.1	(-5.7;-0.5)	0.025	Falling
50-59	46.6	0.8	(-2.7;1.0)	0.332	Stationary
Northeast					
20-29	9.5	-3.4	(-6.4;-0.4)	0.031	Falling
30-39	43.5	-2.4	(-6.3;1.7)	0.227	Stationary
40-49	92.5	-2.7	(-5.8;0.5)	0.091	Stationary
50-59	121.3	0.1	(-3.2;3.7)	0.916	Stationary
Southeast					
20-29	3.9	-5.8	(-11.0;0.7)	0.077	Stationary
30-39	23.1	-3.2	(-7.3;1.0)	0.122	Stationary
40-49	59.5	-2.3	(-6.1;1.5)	0.209	Stationary
50-59	82.8	0.1	(-3.5;3.8)	0.957	Stationary
South					
20-29	3.7	-7.4	(-10.0;-4.3)	<0.001	Falling
30-39	24.4	-3.2	(-7.8;1.5)	0.160	Stationary
40-49	68.1	-2.1	(-3.8;-0.4)	0.021	Falling
50-59	103.1	1.3	(-3.1;5.9)	0.527	Stationary
Midwest					
20-29	6.3	-5.0	(-11.0;1.4)	0.114	Stationary
30-39	34.3	-3.8	(-7.4;-0.1)	0.048	Falling
40-49	77.8	-2.9	(-5.5;-0.3)	0.029	Falling
50-59	103.2	0.6	(-2.2;3.6)	0.631	Stationary

a) APC: Annual percentage change; b) 95%CI: 95% confidence interval; c) p-value: Significance of coeffcient association using Prais-Winsten *t* test regression analysis; d) Stationary trend, when p-value ≥ 0.05, falling when p-value < 0.05 and regression coefficient is negative.

## DISCUSSION

This study identified a stationary trend in the number of deaths from mental and behavioral disorders due to use of alcohol, for Brazil as a whole. The male population accounted for the majority of deaths, the highest proportion among age groups corresponding to those aged 50-59, and more than half of the total self-reported themselves as being of mixed race/skin color. Analysis by region showed a falling trend in individuals aged 20-29 (Northeast and South), 30-39 (Midwest) and 40-49 (North, South and Midwest).

Deaths resulting from mental and behavioral disorders caused by use of alcohol are a constitute condition faced worldwide. Taking Brazil as a reference, between 2010 and 2021, there were more deaths from this disease than from tuberculosis, mainly among people of the male sex, aged 50-59 and of mixed race/skin color.

Predominance of deaths among men has also been found in other studies. The male sex is also related to a higher frequency of hospitalizations resulting from abusive consumption of alcoholic beverages,^4^ ^),(^ ^6^ a possible reflection of the greater and more frequent habit of alcoholic beverage consumption among men. Men start drinking at an earlier age, while there is social and cultural repression for women to not drink excessively. However, a significant increase in the consumption of this substance among women has been seen in recent years.^2^

In our study, deaths were concentrated among Black people (people of Black and mixed race/skin color). This finding corroborates data from another study, which investigated the distribution of deaths attributable to alcohol in Brazil and revealed that 60.0% of them occurred among people of Black and mixed race/skin color.^6^ Another study that analyzed abusive alcohol consumption, based on National Health Survey data, also showed a higher prevalence of consumption among people of Black race/skin color.^2^

These race/skin color indicator results point to an influential factor regarding the poorer health condition of Black people, compared to White people. Historically, the Black population has been more vulnerable to conditions of social and economic inequality, exposed to an overload of risk factors, such as excessive alcohol consumption, sometimes cited as an “escape valve” for these difficulties, which can increase the risk of susceptibility to diseases and harm caused by alcohol abuse.^11^

As for age group, in three regions of Brazil, individuals aged 50-59 were those who died most as a result of mental and behavioral disorders due to use of alcohol. According to overall mortality data from the 2015 “Saúde Brasil” health survey, there was a tendency for mortality coeffcients to increase in this age group,^11^ which could directly affect the country’s economy: this is an age at which people generally are still economically active. On this point, it is worth noting that in 2021 life expectancy at birth in Brazil was 77 years.^6^

The average age for first alcohol intake in Brazil is 12.5 years. The earlier such consumption begins, the greater the likelihood of developing harmful patterns, such as dependence.^12^ ^),(^ ^7^ ^),(^ ^13^ When uncontrolled, excessive alcohol intake can contribute to the emergence of comorbidities in the long term and, consequently, death in later life.

In Brazil, the trend of deaths from to mental and behavioral disorders due to use of alcohol varied between age groups, especially in the 20-29 year group, and also between the country’s macro-regions. The decrease in the number of deaths among people aged 20-29 has continued to occur over the last 20 years in Brazil. Between 2000 and 2013, this age group accounted for only 3.9% of deaths with underlying and/or associated causes fully attributable to alcohol use.^8^ There was also a 4.0% reduction in alcohol consumption among people in the 20-29 age group between 2013 and 2019, when comparing the rates for these years.^6^ From 2010 to 2020, the rate of mental and behavioral disorder hospitalizations due to alcohol use by individuals aged 20-29 was also low, in comparison with such hospitalizations in the other age groups analyzed.^5^

Such results can be attributed to a combination of factors, such as (i) younger people seeking to have a healthier lifestyle, (ii) affirmation of individual identities, (iii) concern about the future and (iv) social pressures.^14^

Furthermore, it is important to highlight that, among the four regions of the country that showed a falling trend in deaths from mental and behavioral disorders due to use of alcohol, the Midwest and Southern regions showed a falling trend for two different age groups.

Similarly to this finding, studies that analyzed mortality due to alcohol dependence and spatial distribution of deaths attributable to use of this substance^4^ ^),(^ ^6^ in the Brazilian population showed that the Southern region of Brazil had the lowest mortality rate in relation to the other regions of the country. Between 2010 and 2020 the Midwest region showed a falling trend in hospitalization rates for mental and behavioral disorders due to use of alcohol.^5^

The falling trend seen in these regions may be related to legislation and strategies to prevent and reduce harm from alcoholic beverages and other drugs in Brazil, such as the prohibition of the sale of alcoholic beverages to minors under 18 years of age, as per article 243 of the Child and Adolescent Statute (Law No. 8069/1990) and by article 63 of the Criminal Misdemeanors Law; and by Law No. 11705/2008, known as the Dry Law. These laws have had a positive impact on the number of hospitalizations resulting from alcohol consumption, contributing to a reduction in the mortality rate due to alcohol-related disorders.^15^

It is noteworthy that the Southeast and Northeast regions, despite having shown a stationary trend in mortality from mental and behavioral disorders due to use of alcohol, were the regions with most records of this condition.

Regarding the limitations of this research, the quality of the Mortality Information System stands out, given the possibility of underrecording of deaths, problems of duplicity and reliability of Death Certificate information, widely reported in the literature,^16^ which may also underestimate mortality from mental and behavioral disorders due to use of alcohol, thus compromising interpretation of trends found. However, these limitations did not invalidate the findings of this study in terms of informing public policy implementation, with emphasis on geographically specific actions, prioritizing territories where high mortality rates due to alcohol use were found.

We conclude that, although the mortality trend has remained stationary in Brazil, it presents variations in its distribution across the country’s major geographic regions, especially with regard to age group. We recommend that further studies be carried out that take into account other variables, such as education, occupation and family income, paving the way for proposition of new explanatory hypotheses for the trend found in the mortality rate from mental and behavioral disorders due to use of alcohol in Brazil.

## References

[1] World Health Organization (2018). Global status report on alcohol and health 2018.

[2] Garcia LP, Freitas LRS (2015). Consumo abusivo de álcool no Brasil: resultados da Pesquisa Nacional de Saúde 2013. Epidemiol Serv Saude.

[3] Rehm J, Mathers C, Popova S, Thavorncharoensap M, Teerawattananon Y, Patra J (2009). Global burden of disease and injury and economic cost attributable to alcohol use and alcohol-use disorders. Lancet.

[4] Oliveira RSC, Matias JC, Fernandes CAOR, Gavioli A, Marangoni SR, Assis FB (2023). Internações por transtornos mentais e comportamentais devidos ao uso de álcool no Brasil e regiões: análise de tendência temporal, 2010-2020. Epidemiol Serv Saude.

[5] Wells RHC, Bay-Nielsen H, Braun R, Israel RA, Laurenti R, Maguin P (2011). CID-10: classificação estatística internacional de doenças e problemas relacionados à saúde.

[6] Marques MV, Silva DN, Santos EGO, Santos SSAN, Neves SMB, Amador AE (2020). Distribuição espacial das mortes atribuíveis ao uso de álcool no Brasil. JHBS.

[7] Antunes JLF, Cardoso MRA (2015). Uso da análise de séries temporais em estudos epidemiológicos. Epidemiol Serv Saude.

[8] Halpern SC, Scherer JN, Roglio V, Faller S, Sordi A, Ornell F (2017). Vulnerabilidades clínicas e sociais em usuários de crack de acordo com a situação de moradia: um estudo multicêntrico de seis capitais brasileiras. Cad Saude Publica.

[9] Ministério da Saúde (BR). Secretaria de Vigilância em Saúde, Ministério da Saúde (BR) (2015). Saúde Brasil 2014: uma análise da situação de saúde e das causas externas.

[10] Instituto Brasileiro de Geografia e Estatística (2022). Tábuas completas de mortalidade em ano de pandemia de COVID-19.

[11] Santana CJ, Oliveira MLF, Martins EAP, Silva AS, Radovanovic CAT, Elvira IKS (2023). Morbimortalidade e fatores associados ao óbito em internados por efeitos do álcool e outras drogas. Esc Anna Nery.

[12] Marín-León L, Oliveira HB, Botega NJ (2007). Mortalidade por dependência de álcool no Brasil: 1998 - 2002. Psicol Estud.

[13] Freitas MG, Stopa SR, Silva EN (2023). Consumo de bebidas alcoólicas no Brasil: estimativa de razões de prevalências - 2013 e 2019. Rev Saude Publica.

[14] Vashishtha R, Pennay A, Dietze P, Marzan MB, Room R, Livingston M (2021). Trends in adolescent drinking across 39 high-income countries: exploring the timing and magnitude of decline. Eur J Public Health.

[15] Ministério da Saúde (BR). Secretaria de Vigilância em Saúde. Departamento de Análise de Situação de Saúde (2011). Plano de ações estratégicas para o enfrentamento das doenças crônicas não transmissíveis (DCNT) no Brasil 2011-2022.

[16] Barbosa JS, Tartaro L, Vasconcelos LDR, Nedel M, Serafini JF, Svirski SGS (2023). Assessment of incompleteness of Mortality Information System records on deaths from external causes in the state of Rio Grande do Sul, Brazil, 2000-2019. Epidemiol Serv Saude.

