# Supplementary material for: Trend in mortality from mental and behavioral disorders due to alcohol use in Brazil, 2010-2021
Source: Epidemiol Serv Saude. 2024 Jul 15;33:e20231483. doi: 10.1590/S2237-96222024V33E20231483.en (PMC11251453; doi:10.1590/S2237-96222024V33E20231483.en)
Supplement: Translation

# Translation: Tendência da mortalidade por transtornos mentais e comportamentais devidos ao uso de álcool no Brasil, 2010-2021

**DOI:** 10.1590/S2237-96222024V33E20231483.pt

**Authors:** Wygor Bruno e Silva Morais, Fernando Ferraz do Nascimento, Jardeliny Corrêa da Penha, Jesusmar Ximenes Andrade, Márcio Dênis Medeiros Mascarenhas, Malvina Thais Pacheco Rodrigues

**Affiliations:** 1 Universidade Federal do Piauí, Programa de Pós-Graduação em Saúde e Comunidade, Teresina, PI, Brasil

**Keywords:** Transtornos Relacionados ao Uso de Álcool, Mortalidade, Alcoolismo, Estudos de Séries Temporais, Saúde do Adulto, Saúde Mental

## Abstract

**Objetivo:**

Analisar a tendência da mortalidade por transtornos mentais e comportamentais devidos ao uso de álcool no Brasil em 2010-2021.

**Métodos:**

Estudo de séries temporais, com dados do Sistema de Informação sobre Mortalidade. A variação percentual anual (VPA) e respectivos intervalos de confiança de 95% (IC_95%_) foram calculados por regressão linear de Prais-Winsten.

**Resultados:**

Houve tendência estacionária na mortalidade no Brasil como um todo (VPA = 0,6; IC_95%_ -4,2;3,0), tendência decrescente em indivíduos de 20-29 anos nas regiões Sul (VPA = -7,4; IC_95%_ -10,0;-4,3) e Nordeste (VPA = -3,4; IC_95%_ -6,4;-0,4), em pessoas de 30-39 anos no Centro-Oeste (VPA = -3,8; IC_95%_ -7,4;-0,1) e naqueles com 40-49 anos nas regiões Sul (VPA = -2,1; IC_95%_ -3,8;-0,4), Norte (VPA = -3,1; IC_95%_ -5,7;-0,5) e Centro-Oeste (VPA = -2,9; IC_95%_ -5,5;-0,3).

**Conclusão:**

A mortalidade por transtornos mentais e comportamentais devidos ao uso de álcool apresentou tendência estacionária no Brasil e decrescente em algumas faixas etárias.

## Contribuições do estudo

**Principais resultados:**

O coeficiente de mortalidade foi maior entre 50-59 anos. Houve tendência estacionária na mortalidade no Brasil como um todo, decrescente em indivíduos de 20-29 (Sul; Nordeste), 30-39 (Centro-Oeste) e 40-49 anos (Norte; Centro-Oeste; Sul).

**Implicações para os serviços:**

Os transtornos mentais e comportamentais devidos ao uso de álcool acometem os brasileiros nas diversas fases da vida, sendo indispensável despertar a atenção da população para a redução do consumo de bebidas alcoólicas.

**Perspectivas:**

É necessário direcionar ações de promoção da saúde e prevenção da mortalidade relacionada ao uso de álcool, com ênfase no grupo etário de 50 a 59 anos.

## INTRODUÇÃO

O álcool é uma droga psicoativa, legalizada e amplamente divulgada, que age diretamente no sistema nervoso central causando dependência química e física.^1^ O uso nocivo dessa substância é um problema de saúde pública, provoca adoecimento, incapacidades e mortes, e está associado ao aparecimento de mais de 200 doenças e lesões, tais como distúrbios mentais, comportamentais e doenças infecciosas, a exemplo da tuberculose e da infecção pelo vírus HIV. Além disso, provoca lesões não intencionais e intencionais, como as causadas por acidentes de trânsito, violência e suicídios, implicando problemas sociais e econômicos para os indivíduos e a sociedade em geral.^2^

Em todo o mundo, a cada ano, aproximadamente 3 milhões de óbitos devem-se ao uso nocivo do álcool, o que corresponde a 5,3% do total de mortes. Com base nos cálculos de anos de vida perdidos ajustados por incapacidade, 5,1% da carga mundial de doenças e lesões são atribuídas ao consumo de álcool. No Brasil, onde cerca de 6,2% dos óbitos estão relacionados ao consumo de bebidas alcoólicas, o álcool ocupa o terceiro lugar entre os fatores de risco para carga de doenças, e o quarto em nível global.^3^

Os transtorno mentais e comportamentais associados ao consumo excessivo de álcool contribuem para a continuada morbimortalidade e incapacidades decorrentes de seu uso nocivo,^3^ ^),(^ ^4^ destacando-se a intoxicação aguda, o uso nocivo para a saúde, a síndrome de dependência, a síndrome de abstinência, a síndrome de abstinência com *delirium* ou estado confusional agudo, o transtorno psicótico, a síndrome amnésica, o transtorno psicótico residual ou de instalação tardia, outros transtornos mentais ou comportamentais e o transtorno mental ou comportamental não especificado.^5^

Estudos brasileiros sobre internações e mortes relacionadas ao consumo do álcool trazem informações relevantes para a compreensão de seus impactos econômicos e sociais na população.^6^ ^)-(^ ^8^ Contudo, a mortalidade por transtornos mentais e comportamentais constitui temática pouco explorada, carente de trabalhos que a dimensionem entre adultos brasileiros, principais consumidores de álcool em excesso no país.

O objetivo do estudo foi analisar a tendência da mortalidade por transtornos mentais e comportamentais devidos ao uso de álcool no Brasil, no período de 2010 a 2021.

## MÉTODOS

*Delineamento*

Estudo de séries temporais, dirigido a analisar a tendência da mortalidade por transtornos mentais e comportamentais decorrentes do uso de álcool no Brasil, no período de 2010 a 2021. As unidades de análise foram as cinco macrorregiões geográficas do país: Norte, Nordeste, Sudeste, Sul e Centro-Oeste.^4^

*Contexto*

O Brasil, maior país da América Latina e quinto no mundo, em área territorial, contava com população de 203 milhões de habitantes em agosto de 2022, distribuídos entre 5.571 municípios de 26 estados e o Distrito Federal.

*Participantes*

Foram incluidos no estudo óbitos de adultos de 20 a 59 anos cuja causa básica de morte tivesse sido “transtornos mentais e comportamentais devidos ao uso de álcool”, ocorridos no Brasil, no período de 2010 a 2021.

*Variáveis*

As variáveis utilizadas no estudo foram:

a) Sociodemográficas

- sexo (masculino; feminino);

- faixa etária (em anos: 20 a 29; 30 a 39; 40 a 49; 50 a 59);

- raça/cor da pele (branca; preta; parda; amarela/indígena);

- macrorregião geográfica do país (Norte; Nordeste; Sudeste; Sul; Centro-Oeste);

- ano do óbito, no período de 2010 a 2021; e

b) Indicador de mortalidade

- coeficiente de mortalidade por transtornos mentais e comportamentais devidos ao uso de álcool.

*Fontes de dados e mensuração*

Os dados de mortalidade foram extraídos da base do Sistema de Informação sobre Mortalidade (SIM), disponível no sítio eletrônico do Departamento de Informática do Sistema Único de Saúde (https://datasus.saude.gov.br/), no período de junho a julho de 2023.

Foram obtidos dados sobre os óbitos ocorridos no período de 2010 a 2021 em residentes nas cinco macrorregiões do Brasil, cuja causa básica de morte estivesse classificada com o código F-10 (Transtornos mentais e comportamentais devidos ao uso de álcool), da Classificação Estatística Internacional de Doenças e Problemas Relacionados à Saúde - Décima Revisão (CID-10). Os dados populacionais, disponibilizados pelo Instituto Brasileiro de Geografia e Estatística (IBGE), foram captados por macrorregião, faixa etária e sexo, relacionados ao período 2000-2021.

Para o cálculo dos coeficientes de mortalidade por transtornos mentais e comportamentais devidos ao uso de álcool, dividiu-se o número de óbitos segundo o local de residência (numerador) no período estudado, pela população do mesmo período e local de residência (denominador), multiplicando-se o quociente por 100 mil habitantes. Estes coeficientes foram calculados por faixa etária, sexo, raça/cor da pele e macrorregião geográfica, para cada ano do óbito. Em seguida foi calculada a média aritmética, somando-se os coeficientes de cada ano e dividindo-os pelo total de 12 anos observados. Para fins de controle do viés de agregação, os dados sobre a população estudada foram estratificados por macrorregião, sexo, faixa etária e raça/cor da pele, para melhor se caracterizar a mortalidade mediante comparação mais homogênea da população.

*Metodos estatísticos*

Os dados foram agrupados em planilhas da plataforma Microsoft Excel e exportados para o programa Statistical Package for the Social Sciences (SPSS), versão 2.0, utilizado nas análises estatísticas. Na verificação da tendência temporal, utilizou-se o modelo de regressão linear de Prais-Winsten, por ser o método indicado para corrigir a autocorrelação serial e permitir a análise de séries temporais acima de nove anos de estudo.^9^ Como variável dependente (Y), adotou-se o coeficiente de mortalidade por transtornos mentais e comportamentais devidos ao uso de álcool, segundo faixa etária, sexo, raça/cor da pele e macrorregião geográfica; e como variável independente (X), o ano de ocorrência do óbito, compreendido no período de 2010 a 2021.

Foi calculado o logaritmo de base 10 dos valores de Y, seguido da aplicação do modelo autorregressivo de Prais-Winsten, para estimar os valores do coeficiente β1 da equação de regressão.

O cálculo da variação percentual anual (VPA) foi realizado a partir da fórmula:

VPA = (-1+10 [b1])*100%

e respectivos intervalos de confiança de 95% (IC_95%_), estes obtidos com a aplicação das seguintes fórmulas:

IC_95%_ mínimo = (-1+10[b1-t*e])*100%

IC_95%_ máximo =(-1+10[b1+t*e ])*100%

Adotam-se o nível de significância de 5%.

A tendência dos coeficientes de mortalidade foi interpretada da seguinte forma: tendência crescente, quando a VPA foi significativamente positiva; decrescente, quando a VPA foi significativamente negativa; e tendência estacionária, quando não houve diferença significativa entre o valor da VPA e zero.^6^

*Aspectos éticos*

O estudo utilizou dados secundários disponíveis em base de dados oficiais do Ministério da Saúde do Brasil, sem identificação de sujeitos. Não foi necessária, portanto, submissão à apreciação de Comitê de Ética em Pesquisa, conforme recomenda a Resolução do Conselho Nacional de Saúde n^o^ 466, de 12 de dezembro de 2012.

## RESULTADOS

No período de 2010 a 2021, o Brasil registrou 60.513 óbitos por transtornos mentais e comportamentais devidos ao uso de álcool. A região Sudeste apresentou o maior número de óbitos em decorrência desse agravo (22.910), enquanto a região Norte (2.056) registrou o menor números de mortes. A maioria dos óbitos correspondeu a indivíduos do sexo masculino (90,0%), e a maior participação foi de indivíduos na idade dos 50 aos 59 anos (40,6%) e autodeclarados pardos (50,3%) (Tabela 1).

**Tabela 1:** Caracterização sociodemográfica dos óbitos por transtornos mentais e comportamentais devidos ao uso de álcool em indivíduos de 20 a 59 anos de idade, Brasil, 2010-2021

Variável	Norte		Nordeste		Centro-Oeste		Sul		Sudeste		Brasil	
	N (2.056)	%	N (21.120)	%	N (5.244)	%	N (9.183)	%	N (22.910)	%	N (60.513)	%
Sexo												
Masculino	1.858	90,4	19.260	91,2	4.705	89,7	8.437	92	20.263	88,5	54.523	90,0
Feminino	198	9,6	1.860	8,8	539	10,3	746	8,0	2.647	11,5	5.990	10,0
Raça/cor da pele												
Branca	217	10,5	2.691	12,7	1.180	22,5	6.481	70,5	8.408	37,0	18.977	31,4
Parda	1.540	75,0	14.660	69,5	3.241	62,0	1.675	18,0	9.333	40,7	30.449	50,3
Preta	194	9,5	2.477	11,7	616	11,5	772	8,5	4.044	17,5	8.103	13,3
Amarela e indigena	41	2,0	108	0,5	107	2,0	69	1,0	70	0,3	395	0,7
Ignorada	64	3, 0	1.184	5,6	100	2,0	186	2,0	1.055	4,5	2.589	4,3
Faixa etária (anos)												
20-29	122	6,0	1.129	5,3	206	4,0	215	2,0	656	3,0	2.328	3,8
30-39	438	21,5	4.585	21,7	1.078	20,5	1.318	14,5	3.852	17,0	11.271	18,6
40-49	731	35,5	7.767	36,8	1.999	38,0	3.301	36,0	8.519	37,0	22.317	37,0
50-59	765	37,0	7.640	36,2	1.961	37,5	4.349	47,5	9.888	43,0	24.603	40,6

O coeficiente de mortalidade apresentou-se mais elevado no grupo etário de 50 a 59 anos, no Brasil como um todo e em cada uma de suas macrorregiões. No conjunto do país, o coeficiente de mortalidade para o grupo de 50 a 59 anos variou de 10,1 a 11,3 por 100 mil hab. entre 2010 e 2021, indicativo de tendência estacionária (VPA = 0,5; IC_95%_ -3,1;4,2). As demais faixas de idade apresentaram redução do coeficiente, tanto para a totalidade do país como para cada uma de suas regiões (Figura 1).

**Figura 1:** Coeficiente de mortalidade por transtornos mentais e comportamentais devidos ao uso de álcool em indivíduos de 20 a 59 anos de idade, por macrorregiões nacionais, Brasil, 2010-2021

Observou-se tendência estacionária no coeficiente de mortalidade por transtornos mentais e comportamentais devidos ao uso de álcool no Brasil como um todo (VPA = 0,6; IC_95%_ -4,2;3,0), em ambos os sexos e nas cinco regiões (Tabela 2). A tendência dos coeficientes de mortalidade segundo a faixa etária, entretanto, divergiu entre as regiões. Ela foi decrescente para pessoas com 20 a 29 anos residentes nas regiões Nordeste (VPA = -3,4; IC_95%_ -6,4;-0,4) e Sul (VPA = -7,4; IC_95%_ -10,0;-4,3), enquanto as regiões Norte (VPA = -4,5; IC_95%_ -11,0;2,4), Sudeste (VPA = -5,8; IC_95%_ -11,0;0,7) e Centro-Oeste (VPA = -5,0; IC_95%_ -11,0;1,4) apresentaram tendência estacionária para a mesma faixa de idade. Ao se observarem aqueles com 30 a 39 anos, somente a região Centro-Oeste mostrou tendência decrescente na mortalidade pelos mesmos transtornos mentais e comportamentais devidos ao álcool (VPA = -3,8; IC_95%_ -7,4;-0,1). Entre os indivíduos de 40 a 49 anos, essa tendência foi decrescente nas regiões Norte (VPA = -3,1; IC_95%_ -5,7;-0,5), Sul (VPA = -2,1; IC_95%_ -3,8;-0,4) e Centro-Oeste (VPA = -2,9; IC_95%_ -5,5;-0,3) (Tabela 3). A mortalidade entre indivíduos na idade de 50 a 59 anos apresentou tendência estacionária nas cinco regiões do país (Figura 1).

**Tabela 2:** Tendência dos coeficientes de mortalidade por transtornos mentais e comportamentais devidos ao uso de álcool em indivíduos de 20 a 59 anos de idade, por macrorregião geográfica e sexo, Brasil, 2010-2021

Variables	Coeficiente médio	VPA^a^ (%)	IC_95%_^b^	p-valor^c^	Interpretação^d^
Região					
Brasil	41,4	0,6	(-4,2;3,0)	0,694	Estacionária
Norte	18,2	-1,7	(-3,7;0,4)	0,111	Estacionária
Nordeste	56,9	-0,8	(-4,2;2,7)	0,606	Estacionária
Sudeste	38,3	0,8	(-4,7;3,3)	0,670	Estacionária
Sul	45,5	0,1	(-3,7;4,2)	0,928	Estacionária
Centro-Oeste	48,3	-0,9	(-3,8;1,9)	0,474	Estacionária
Sexo masculino					
Brasil	76,5	-0,6	(3,2;-4,2)	0,728	Estacionária
Norte	32,8	2,0	(-4,0;0,0)	0,060	Estacionária
Nordeste	107,6	0,7	(-4,3;3,0)	0,682	Estacionária
Sudeste	69,4	-0,6	(-4,7;3,3)	0,741	Estacionária
Sul	85,0	-0,0	(-3,6;3,8)	0,995	Estacionária
Centro-Oeste	87,8	-0,9	(-4,0;2,2)	0,517	Estacionária
Sexo feminino					
Brasil	7,7	-1,7	(-4,2;0,9)	0,173	Estacionária
Norte	3,4	1,2	(-2,0;4,6)	0,435	Estacionária
Nordeste	9,6	-1,8	(-3,9;0,3)	0,095	Estacionária
Sudeste	8,6	-2,4	(-4,9;0,1)	0,061	Estacionária
Sul	7,3	-1,8	(-5,7;2,3)	0,335	Estacionária
Centro-Oeste	9,8	-1,8	(-4,8;1,2)	0,205	Estacionária

a) VPA: Variação percentual anual; b) IC_95%_: Intervalo de confiança de 95%; c) p-valor: Significância da associação dos coeficientes pelo teste *t* de regressão de Prais-Winsten; d) Tendência estacionária quando p-valor ≥ 0,05.

**Tabela 3:** Tendência dos coeficientes de mortalidade por transtornos mentais e comportamentais devidos ao uso de álcool em indivíduos de 20 a 59 anos de idade, por região e faixa etária, Brasil, 2010-2021

Faixa etária (anos)	Coeficiente médio	VPC^a^ (%)	IC_95%_^b^	p-valor^c^	Interpretação^d^
Brasil					
20-29	5,5	-4,2	(-7,8;-0,2)	0,038	Decrescente
30-39	27,7	-2,7	(-6,8;1,7)	0,199	Estacionária
40-49	65,6	-2,5	(-5,5;0,6)	0,103	Estacionária
50-59	91,5	0,5	(-3,1;4,2)	0,762	Estacionária
Norte					
20-29	3,1	-4,5	(-11,0;2,4)	0,174	Estacionária
30-39	13,2	-3,1	(-8,1;2,2)	0,222	Estacionária
40-49	30,1	-3,1	(-5,7;-0,5)	0,025	Decrescente
50-59	46,6	0,8	(-2,7;1,0)	0,332	Estacionária
Nordeste					
20-29	9,5	-3,4	(-6,4;-0,4)	0,031	Decrescente
30-39	43,5	-2,4	(-6,3;1,7)	0,227	Estacionária
40-49	92,5	-2,7	(-5,8;0,5)	0,091	Estacionária
50-59	121,3	0,1	(-3,2;3,7)	0,916	Estacionária
Sudeste					
20-29	3,9	-5,8	(-11,0;0,7)	0,077	Estacionária
30-39	23,1	-3,2	(-7,3;1,0)	0,122	Estacionária
40-49	59,5	-2,3	(-6,1;1,5)	0,209	Estacionária
50-59	82,8	0,1	(-3,5;3,8)	0,957	Estacionária
Sul					
20-29	3,7	-7,4	(-10,0;-4,3)	<0,001	Decrescente
30-39	24,4	-3,2	(-7,8;1,5)	0,160	Estacionária
40-49	68,1	-2,1	(-3,8;-0,4)	0,021	Decrescente
50-59	103,1	1,3	(-3,1;5,9)	0,527	Estacionária
Centro-Oeste					
20-29	6,3	-5,0	(-11,0;1,4)	0,114	Estacionária
30-39	34,3	-3,8	(-7,4;-0,1)	0,048	Decrescente
40-49	77,8	-2,9	(-5,5;-0,3)	0,029	Decrescente
50-59	103,2	0,6	(-2,2;3,6)	0,631	Estacionária

a) VPA: Variação percentual anual; b) IC_95%_: Intervalo de confiança de 95%; c) p-valor: Significância da associação dos coeficientes pelo teste *t* de regressão de Prais-Winsten; d) Tendência estacionária, quando p-valor ≥ 0,05, decrescente quando p-valor < 0,05 e o coeficiente da regressão negativo.

## DISCUSSÃO

Este estudo identificou tendência estacionária do número de óbitos por transtornos mentais e comportamentais devidos ao uso de álcool, para o Brasil como um todo. A população masculina concentrou a maioria dos óbitos, cuja maior proporção entre as faixas etárias correspondeu aos 50-59 anos, e mais da metade do total foi de autodeclarados pardos. A análise por regiões mostrou tendência decrescente em indivíduos com idade de 20 a 29 (Nordeste e Sul), 30 a 39 (Centro-Oeste) e 40 a 49 anos (Norte, Sul e Centro-Oeste).

Os óbitos decorrentes de transtornos mentais e comportamentais causados pelo uso de álcool constituem condição a ser enfrentada no mundo. Tomando como referência o Brasil, entre os anos de 2010 e 2021, ocorreram mais óbitos por esse agravo do que por tuberculose, principalmente entre pessoas do sexo masculino, idade de 50 a 59 anos e raça/cor da pele parda.

A predominância de óbitos em homens foi observada em outros estudos, além deste. O sexo masculino também está relacionado a maior frequência de internações decorrentes do consumo abusivo de bebidas alcoólicas,^4^ ^),(^ ^6^ possível reflexo do hábito de consumir bebida alcoólica entre os homens, maior e mais frequente. Homens começam a beber com menor idade, enquanto há repressão social e cultural para que as mulheres não bebam em excesso. Não obstante, foi observado aumento expressivo no consumo dessa substância entre mulheres, nos últimos anos.^2^

Na presente análise, os óbitos concentraram-se em pessoas negras (pretas e pardas). Este achado corrobora os dados de outro estudo, que investigou a distribuição de óbitos atribuíveis ao álcool no Brasil e revelou serem 60,0% deles de pessoas com raça/cor da pele preta e parda.^6^ Um estudo que analisou o consumo abusivo de álcool, dessa vez com dados da Pesquisa Nacional de Saúde (PNS), também evidenciou maior prevalência de consumo entre pessoas negras.^2^

Esses resultados do indicador raça/cor da pele apontam para fator influente na pior condição de saúde dos negros, comparados aos brancos. Historicamente, a população negra está mais vulnerável a condições de desigualdade social e econômica, exposta a sobrecarga de fatores de risco, como o consumo excessivo de bebida alcoólica, por vezes citado como “válvula de escape” dessas dificuldades, o que pode aumentar a susceptibilidade às doenças e agravos provocados pelo uso abusivo do álcool.^11^

Quanto à faixa etária, em três regiões do país, indivíduos na idade dos 50 aos 59 anos foram os que mais morreram em decorrência de transtornos mentais e comportamentais devidos ao uso de álcool. Segundo os dados gerais de mortalidade da pesquisa Saúde Brasil de 2015, houve tendência de aumento dos coeficientes de mortalidade nessa faixa etária,^11^ o que pode afetar diretamente a economia do país: trata-se de idade na qual as pessoas, geralmente, continuam ativas; cabe lembrar que, em 2021, a expectativa de vida ao nascer no Brasil era de 77 anos.^6^

A idade média para o primeiro consumo de álcool no Brasil é de 12,5 anos. Quanto mais precoce o início desse consumo, maior a probabilidade de desenvolver padrões nocivos, como dependência.^12^ ^),(^ ^7^ ^),(^ ^13^ Quando não controlada, a ingestão excessiva de bebidas alcoólicas pode contribuir para o aparecimento de comorbidades no longo prazo e, consequentemente, o óbito em idade mais avançada.

No Brasil, a tendência de óbitos em decorrência de transtornos mentais e comportamentais devidos ao uso de álcool sofreu variação entre as faixas etárias, especialmente de 20 a 29 anos, e entre as grandes regiões nacionais. O decréscimo do número de óbitos entre pessoas de 20 a 29 anos vem se mantendo ao longo dos últimos 20 anos, no país. Entre 2000 e 2013, essa faixa etária correspondeu a tão somente 3,9% dos óbitos com causa básica e/ou associada, plenamente atribuível ao uso de álcool.^8^ Houve também redução de 4,0% no consumo de álcool entre pessoas na faixa etária de 20 a 29 anos, em 2013 e 2019, quando comparados os valores de um ano com o outro.^6^ De 2010 a 2020, o coeficiente de internações para atendimento a transtornos mentais e comportamentais devidos ao uso de álcool por indivíduos de 20 a 29 anos também foi baixo, na comparação com essas internações nas demais faixas analisadas.^5^

Tais resultados podem ser atribuídos a uma combinação de fatores, como busca por estilo de vida saudável entre as pessoas mais jovens, afirmação de identidades individuais, preocupação com o futuro e pressões sociais.^14^

Ademais, é importante ressaltar que, entre as quatro regiões do país que apresentaram tendência de óbitos em decorrência de transtornos mentais e comportamentais devidos ao uso de álcool, o Centro-Oeste e o Sul apresentaram tendência decrescente para duas faixas etárias distintas.

Semelhantemente a esse achado, estudos que analisaram a mortalidade por dependência de álcool e a distribuição espacial das mortes atribuíveis ao uso dessa substância^4^ ^),(^ ^6^ ^)^ na população brasileira mostraram que o Sul brasileiro apresentou o menor coeficiente de mortalidade em relação às demais regiões do país. Já o Centro-Oeste, entre os anos de 2010 e 2020, apresentou tendência de redução nos coeficientes de internações por transtornos mentais e comportamentais devidos ao uso de álcool.^5^

A tendência decrescente observada nessas regiões pode estar relacionada à legislação e a estratégias de prevenção e redução de danos da bebida alcoólica e de outras drogas no Brasil, como a proibição da venda de bebidas alcoólicas para menores de 18 anos, pelo artigo 243 do Estatuto da Criança e do Adolescente (Lei n^o^ 8.069/90) e pelo artigo 63 da Lei das Contravenções Penais; e pela Lei n^o^ 11.705, de 19 de junho de 2008, conhecida como Lei Seca. Estas leis tiveram impacto positivo sobre o número de internações decorrentes do consumo, colaborando com a redução no coeficiente de mortalidade por transtornos devidos ao álcool.^15^

Destaca-se que as regiões Sudeste e Nordeste, apesar de terem apresentado tendência estacionária de mortalidade em decorrência de transtornos mentais e comportamentais devidos ao uso de álcool, foram as que mais apresentaram registros desse agravo.

Em relação às limitações desta pesquisa, destaca-se a qualidade do SIM, dada a possibilidade de sub-registro de óbitos, problemas de duplicidade e confiabilidade das informações da Declaração de Óbito, amplamente relatados na literatura,^16^ que podem, inclusive, subestimar a mortalidade por transtornos mentais e comportamentais devidos ao uso do álcool, comprometendo a interpretação das tendências observadas. Contudo, essas limitações não inviabilizaram os achados do presente estudo enquanto subsídios à implementação de políticas públicas, com ênfase em ações geograficamente especificas, priorizando territórios que apresentaram elevados coeficientes de mortalidades em decorrência do uso de álcool.

Conclui-se que, embora tenha se mantido estacionária no país, a tendência de mortalidade apresenta variações em sua distribuição pelas grandes regiões geográficas, principalmente no que se refere à faixa etária. Recomenda-se a realização de mais estudos que levem em consideração outras variáveis, como escolaridade, ocupação e renda familiar, abrindo espaço à proposição de novas hipóteses explicativas para a tendência observada no coeficiente de mortalidade por transtornos mentais e comportamentais devidos ao uso de álcool no Brasil.
